# The Neurometabolic Function of the Dopamine–Aminotransferase System

**DOI:** 10.3390/metabo15010021

**Published:** 2025-01-06

**Authors:** Sergey A. Apryatin

**Affiliations:** Institute of Translational Biomedicine, Saint Petersburg State University, 199034 Saint Petersburg, Russia; s.apryatin@spbu.ru

**Keywords:** neurometabolic function, dopamine–aminotransferase system, aspartate aminotransferase, alanine aminotransferase, tyrosine aminotransferase, De Ritis ratio, CREB transcription factor

## Abstract

Background/Objectives: The neurometabolic function is controlled by a complex multi-level physiological system that includes neurochemical, hormonal, immunological, sensory, and metabolic components. Functional disorders of monoamine systems are often detected in clinical practice together with metabolic dysfunctions. An important part of the mentioned pathological conditions are associated with disturbances in protein metabolism, some of the most important biomarkers which are aminotransferases and transcription factors that regulate and direct the most important metabolic reactions. Another important part of energy metabolism is the dopamine-mediated regulation of protein metabolism. Methods: The review describes research results into the dopamine-mediated mechanism of metabolic regulation in humans and animals. Particular attention is paid to the neurometabolic mechanisms of protein metabolism. Results: The dopamine–aminotransferase system of the energy metabolism regulation is a separate, independent, regulatory and diagnostically significant biochemical pathway controlled by the hormonal system, the key hormone is cortisol, the key neurotransmitter is dopamine, the key transcription factor is CREB, and the key regulatory enzymes are alanine aminotransferase, aspartate aminotransferase, and tyrosine aminotransferase. Conclusions: This review presents an original study describing the discovery of a new regulatory mechanism for neurometabolic physiological function in humans and animals. A key part of this mechanism is the dopamine–aminotransferase system.

## 1. Introduction

Dysfunctions of monoamine neurotransmitter systems lead to serious changes in behavior, cognitive, metabolic, and other pathological conditions [1]. Some of the most important regulators of complex forms of behavioral disorders are dopamine systems, trace amines, and their receptors [2,3,4,5]. As early as the middle of the last century, it was proven that dopamine plays a key role in the transmission of nerve impulses in the brain [4], regulation of motor neurons [6], functioning of the reward system [7], spatial memory [8], motivation [9], arousal [10,11], sexual behavior [12], etc. However, this influence has been shown not only in classical physiological reactions associated, for example, with locomotor activity but also in eating behavior, as well as participation in the regulation of various metabolic reactions. One of the main causes of such dysfunctions is the violation of dopamine reuptake in the synaptic cleft, for which the dopamine transporter (DAT) is responsible [13]. Over the past 20 years, with the discovery of a new family of TAAR receptors [14,15], the important role of trace amines in the regulation of dopamine systems, their connection with functional disorders of monoamine systems and metabolic dysfunctions, including such pathological conditions as Attention Deficit Hyperactivity Disorder (ADHD), Major Depressive Disorder (MDD), Parkinson’s disease, schizophrenia, memory and spatial orientation disorders, obesity, metabolic syndrome, etc., has been studied and proven [5]. Trace amines (TA) are formed in the body as a result of decarboxylation of all amino acids and are metabolites of endogenous monoamine neurotransmitters. TA and the Trace Amine-Associated Receptor family (TAARs) represent a monoamine system that affects dopaminergic and other systems regulating the development of various neurodegenerative processes and metabolic disorders, including signal transmission, neurogenesis, energy metabolism, and other physiological processes [5,16].

Functional disorders of monoamine systems are often detected in clinical practice together with metabolic dysfunctions (obesity, diabetes mellitus, metabolic syndrome, etc.) [17,18,19,20]. Many of these pathological conditions are the cause of disability of patients and lead to high mortality rates.

The lifestyle of modern people—low physical activity, high stress, eating disorders, and imbalances in the diet, for example, increased content of simple sugars, saturated fats in food, a lack of vitamins, and macro- and microelements—is the main reason for the growth of the above mentioned pathological conditions throughout the world [21,22,23]. Increased stress loads and the development of concomitant pathological conditions often lead to eating disorders [24], which are characterized not only by excessive consumption of simple carbohydrates, leading to the gradual development of insulin resistance, but also by functional disorders of dopamine and other neurotransmitter systems [22].

It is necessary to separately note the importance of the genetic component in the development of a complex of pathological conditions associated with lifestyle disorders, poor nutrition, etc. [21]. Genetic predisposition often contributes to the rapid development of the mentioned diseases. At the same time, its immediate consequence is various behavioral and metabolic changes in the patient.

A large amount of scientific information has been accumulated related to the influence of dopamine systems on behavioral and metabolic disorders, which has made it possible to reveal some of the mechanisms of development of the mentioned pathological conditions, including interaction with reward systems, dopamine signal reception, mood regulation, metabolic changes, etc. [1,2,13,25,26]. The connection of the trace amine system with dopamine and other monoamine systems of the brain has been shown; however, these studies have begun to actively develop only in the last 20 years, which is associated with the discovery of the first trace amine receptor, TAAR1 [5,27].

On the other hand, many of the biological mechanisms of the influence of monoamine systems on behavioral and metabolic disorders are currently unknown or have not been sufficiently studied [5,21]. An important part of the mentioned pathological conditions is associated with disturbances in protein metabolism, some of the most important enzymes of which are aminotransferases (aspartate aminotransferase, alanine aminotransferase, tyrosine aminotransferase, etc.), as well as the cyclic adenosine monophosphate (cAMP) response element binding protein (CREB transcription factor) which regulate and direct the most important metabolic reactions [28,29,30].

The most interesting and little-studied link in the above mechanisms is the dopamine–aminotransferase physiological system—a part of protein metabolism that largely determines and directly affects catabolic and anabolic changes in the body, regulated by hormonal and various monoamine neurotransmitter systems, the key neurotransmitter of which is dopamine.

## 2. Monoamine Neurotransmitters

The monoamine system of the brain is a complex neurotransmitter system that performs the most important biological functions of the body mediated by the central nervous system. These include the dopaminergic, serotonergic, and noradrenergic systems, as well as the trace amine system [31].

Monoamine neurotransmitters (dopamine, serotonin, norepinephrine, etc.) have many common biochemical characteristics. They are small charged molecules that usually do not cross the blood–brain barrier, formed from amino acids with the help of decarboxylase enzymes [32]. In general, monoamine neurotransmitters act on neurons by binding to metabotropic receptors and have a slower modulation of signaling compared to glutamate- and GABA-mediated neurotransmission [33], except serotonin, which can also bind to ionotropic receptors [34]. One group of monoamines, the catecholamines, derived from the amino acid tyrosine, includes three main neurotransmitters: norepinephrine, epinephrine, and dopamine [35]. Norepinephrine generally regulates wakefulness and sleep patterns, attention span, and alertness [36,37], while epinephrine controls adrenal function, sleep, and the fight-or-flight response [38,39]. As one of the major monoamine neurotransmitters, dopamine plays an important role in the regulation of motor neuron function [40], spatial memory function [8], motivation [9], arousal [10,41], and reward and pleasure [7], as well as lactation [42], sexual behavior [12], and nausea [43]. Dopamine-producing neurons were first included in the relevant neuronal system maps of the brain in 1964 [44]. In humans, projections of dopaminergic neurons from the substantia nigra pars compacta to the striatum, known as the nigrostriatal pathway, control movement and motor learning [45]. Even partial loss of these dopaminergic neurons leads to the manifestation of symptoms of Parkinson’s disease. Trace amines (TA), such as β-phenylethylamine, p-tyramine, p-octopamine, etc., are formed in the body as a result of the decarboxylation of all known amino acids during the thermal or enzymatic processing of food products, including with the participation of the gastrointestinal tract microflora [29,46] and without it (meat, fish, cocoa, chocolate, cheese, etc.), and are also metabolites of endogenous monoamine neurotransmitters—dopamine, serotonin, norepinephrine, etc. [47,48,49,50].

The first trace amine receptor, TAAR1, was discovered in 2001 [27,51], which served to isolate a separate group of endogenous monoamines that have their independent receptor system involved in the pathogenesis of various diseases [47,52]. To date, more than 400 receptors (products of various genes) of TA are known. Their number in different animal species varies from 0 (in some dolphin species) to 497 (in the *Erpetoichtys calibaricus* fish), while many more different isoforms and splicing variants have been identified. In humans, rats, and mice, six functionally active receptors have been found—TAAR1, 2, 5, 6, 8, 9 and three pseudogenes—TAAR3, 4, and 7 [47].

The term “trace amines” was coined and introduced into scientific practice in the early 1970s by Alan Boulton [53], who wanted to emphasize the low concentration (less than 100 ng/g tissue) of TA in comparison with the concentration of classical neurotransmitters [54]. Trace amines are found not only in thermally or enzymatically processed foods but also in many fresh foods, in mg/kg concentrations range [47,55]. Many biogenic amines, such as tyramine, are present in nanomolar concentrations in blood plasma and in the central nervous system (mainly in neurons) of healthy people [56].

TA are broken down by the enzymes monoamine oxidases A and B (MAO-A and MAO-B). In this case, as a result of the use of MAO inhibitors, the so-called “cheese” syndrome often develops, leading to hypertension and headaches that occur after excessive consumption of foods containing trace amines (cheese, red wine, chocolate, etc.) [29,57].

Until recently, it was believed that, except for TAAR1, all other representatives of this receptor family function primarily as chemosensory receptors in the olfactory system [58]. However, recently, a wide range of biological functions have been demonstrated for a wide range of TAAR receptors, including the regulation of metabolic pathways involved in the pathogenesis of alimentary-dependent, neurodegenerative, oncological, cardiovascular, and other diseases [29,56,59,60]. The mechanisms of TAAR1 receptor activation, as in the case of dopamine, are associated with intracellular signaling via cAMP, phosphorylation of protein kinase A and protein kinase C, and subsequent signal transmission to the nucleus [61]. In the striatum of TAAR1-KO mice, overexpression of both mRNA and protein of the D2 dopamine receptor, but not D1, was detected. The AKT/GSK3 signaling pathway (not associated with the D2 receptor) was selectively activated, which is associated with the phosphorylation of AKT and GSK3β [62]. TAAR1 is currently being studied as a potential therapeutic target for the treatment of various mental disorders, such as schizophrenia [29,63,64,65,66].

TAAR1 expression in the brain of experimental animals is observed predominantly in monoaminergic, in particular dopaminergic, neurons. TAAR1 expression has been studied quite well using functional magnetic resonance imaging in human dopaminergic neurons [29,66], including in various areas of the human brain associated with the dopamine-mediated reward system [62].

In peripheral organs and tissues, TAAR1 has been detected in pancreatic β-cells, where it promotes glucose-dependent insulin secretion, and in the small intestine [67]. TAAR1 enhances glucose-stimulated insulin secretion via cAMP-PKA-dependent signaling in pancreatic β-cells. At the same time, calcium is necessary for TAAR1-MAPK signaling in insulin-secreting cells.

Consumption of a high-fructose diet is associated with effects on brain dopamine metabolism, neuromotor function, and anxiety levels in TAAR1 knockout mice. In a comparative analysis of behavioral, biochemical, and morphological parameters, significant differences were found between integral liver parameters and biochemical parameters responsible for protein metabolism regulation (AST/ALT ratio, creatine kinase activity, and urea) and behavioral changes [68]. Over the past few years, there has been an increase in scientific publications showing good prospects for the trace amine system in biomedicine, including development, and preclinical/clinical studies of drugs, cosmetics, dietary supplements, and specialized food products. The role of TA in controlling behavior, energy metabolism, and cellular immune responses, including interaction with microbiota, in the biochemical transformations of nutrients in the body and, consequently, in the pathogenesis of alimentary-dependent diseases has been shown [29,48].

Trace amines are part of many diets; for example, the Mediterranean diet. Products containing TA (seafood, wine, cheese, and other fermented products) are natural antidepressants [29]. In addition, the connection of TA and its receptors with the dopamine system has become a putative etiological factor in drug abuse, as well as various mental disorders.

Decarboxylation of amino acids (the main endogenous mechanism of TA formation) occurs in the brain with high intensity, which affects the participation of trace amines in the regulation of neurodegenerative processes. However, to determine the molecular mechanisms of the influence of protein nutrients and endogenous products of amino acid decarboxylation on the level of TA in the brain, it is necessary to use knockout (for genes encoding TAARs) animals. In clinical practice, the determination of TA levels in the blood may be diagnostically significant.

## 3. Dopaminergic Systems

In a series of scientific papers, the key one being an article in the journal Science [69], the Swedish scientist Arvid Carlsson demonstrated that dopamine (DA) is an independent brain neurotransmitter. DA is synthesized from the amino acid tyrosine in two stages. In the first stage, L-tyrosine is hydroxylated by the enzyme tyrosine hydroxylase to form L-DOPA. The L-DOPA is decarboxylated by the enzyme L-DOPA decarboxylase to dopamine form in the second stage [70].

Dopamine systems perform important functions in neuromodulation, such as motor control (locomotor activity), motivation, reward, cognitive functions, and maternal and reproductive behavior. Dopamine is synthesized in the central nervous system and the periphery and exerts its effect by binding to G-protein-coupled receptors (GPCRs).

**Figure 1:**
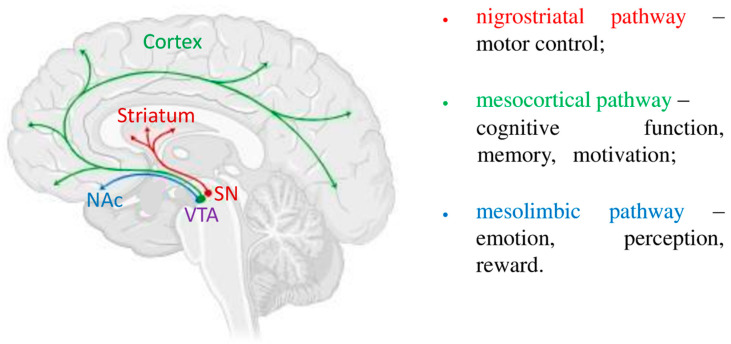
The main dopaminergic signal transmission pathways and their functions. SN—substantia nigra, VTA—ventral tegmental area, NAs—nucleus accumbens.

There are several types of dopamine receptors—D1R, D2R, D3R, D4R, and D5R [70,71,72]. D1R and D5R are part of the D1 subgroup, and D2R, D3R, and D4R are D2 receptors. These receptors are expressed in various tissues and organs of the body, act in the peripheral and central nervous system, and differ in structure and pharmacological properties. Dopaminergic signaling pathways are important for maintaining physiological processes, and unbalanced activity of dopamine systems can lead to dysfunctions associated with neurodegenerative diseases. Uncovering the neurobiology and molecular mechanisms underlying these diseases can contribute to the development of new treatments, which is necessary for improving the quality of life of patients worldwide [72]. The main dopaminergic neurotransmission pathways in the brain are the nigrostriatal, mesocortical, and mesolimbic pathways (Figure 1) [72]. Neurons of the nigrostriatal, mesocortical, and mesolimbic systems of the brain form a complex of neurons in the substantia nigra (SN) and ventral tegmental area (VTA). These areas include projections of these neurons, which partially overlap, creating a continuous cellular network [70,72,73,74].

Approximately 80% of brain dopamine is released by the axons of neurons in the nigrostriatal pathway. These are primarily neurons in the substantia nigra and the lateral part of the ventral tegmental area [72]. Moreover, neurons in the first of these sections project to the dorsal striatum, and neurons in the second section project to the nucleus accumbens (NAc) of the brain [70]. Another equally important dopamine system in the brain is the mesocortical part.

The cell bodies of neurons in this system are located in the VTA and have primary projections to the prefrontal cortex (PFC) [73,74,75,76].

Many studies of dysfunctions associated with the mesolimbic dopaminergic signaling pathway report a surprising variety of behavioral disturbances that can not be explained in detail by the relatively small number of functions of midbrain dopamine neurons [73,74,75,76,77]. To uncover the basic dopamine function underlying these phenomena, electrophysiological and neurochemical studies still provide confusing, mutually exclusive, and partly contradictory explanations for the role of dopamine in behavior. However, the rate of observed phasic changes in dopamine varies up to several thousand-foldthousand-fold, which makes it possible to differentiate changes in behavior.

Projections of neurons in the mesolimbic pathway are mainly associated with the VTA and the substantia nigra. In addition, the axons of neurons of this system are found in the amygdala, hippocampus, nucleus accumbens, cingulate gyrus, olfactory tubercle, parahippocampal gyrus, septum, and other structures of their limbic system of the brain, which has a major influence on the hypothalamus and prefrontal cortex. DA is involved in mediating the body’s reactivity to the environment at various time intervals, from rapid impulse responses associated with reward to slower changes, such as movement, to the activation of postsynaptic motor, cognitive, and motivational systems, which are impaired in Parkinson’s disease.

The generally accepted hypothesis about the influence of dopamine on locomotor function was initially proposed by a neurologist after examining patients with Parkinsonism. Thus, in Parkinson’s disease there is bradykinesia which, when facial muscles are involved, then leads hypomimia. Such patients often suffer from visual impairment, resting tremor and muscle rigidity [78].

Monkeys and rats with pharmacologically reduced dopamine levels, as well as patients with Parkinsonism, have multiple and significant cognitive impairments associated with the deterioration of working memory [79], decision making [80], behavior [81], direction of movement [82], strategy formation [83], attention [84,85], and mental flexibility [86]. Impulsivity, gambling, attention deficit hyperactivity disorder (ADHD), and restless legs syndrome are based on changes in dopamine levels or dopamine receptor polymorphisms [87,88,89]. In contrast, other behavioral functions are less affected, including externally (versus internally) controlled behavior, memory, and learning [90,91]. Injections of dopamine receptor antagonists in monkeys result in changes in working memory function and locomotor activity associated with prefrontal cortex and striatum activity [71,92].

Dopamine systems are significantly involved in mood regulation [25], and the D2 receptor has been widely studied with regard to various mental disorders. However, the antidepressant regulatory function of dopamine systems requires further clarification and pharmacogenetic studies [93]. The association of two polymorphisms rs4460839/rs2734833 of the gene encoding the DRD2 receptor was correlated with the early onset of depressive disorders. Depressive symptoms were detected even after antidepressant treatment [26]. Moreover, DRD4 has a high homology with DRD2; the VNTR in exon 3 can modulate the beneficial effect of antidepressants and predict their therapeutic efficacy [94]. The gene encoding the dopamine transporter (DAT) contains a 40 bp VNTR fragment in exon 15, which is proposed as a genotype that disrupts DAT function. It was found that all patients with the above genotype showed a complete absence of or weak therapeutic effect in response to antidepressant treatment. At the same time, certain positive effects of various antidepressants were found, including tricyclic drugs, mirtazapine, and venlafaxine [95]. As mentioned above, dopamine plays an important role in eating behavior regulation. In the hypothalamus, it increases nutrients consumption [96], which is directly related to the reward system [97]. Consumption of fats and carbohydrates [17] leads to the release of DA in the ventral hypothalamic region, nucleus accumbens, and prefrontal cortex of the hypothalamus. DA-related disorders have been identified in metabolic changes in animals and humans. Consumption of a high-fat diet alters the expression of DA metabolism genes in the hypothalamus [98]. Another interesting effect is the decreased expression of the dopamine D2 receptor within the mesocorticolimbic system in rats and humans with obesity [99], reduced DA concentration in the striatum in rats with alimentary obesity [100], and suppressed expression of the D1 receptor in the nucleus accumbens in rats genetically predisposed to excess weight gain [101], indicating an important regulatory role of decreased DA levels in the brain during the development of alimentary forms of obesity. Dopaminergic neurons of the ventral hypothalamus play an important role in stimulating feeding behavior under the influence of cannabinoids, alcohol, and other psychoactive substances. It is important to note that dopamine-dependent regulation of feeding behavior is mediated by ghrelin and other neuropeptides [102]. Dopamine is a member of the catecholamine family and is associated with a variety of physiological functions. Together with its five receptor subtypes, dopamine is closely associated with neurological disorders such as schizophrenia, Parkinson’s disease, depression, attention deficit hyperactivity disorder, etc. The high degree of cross-interaction of dopamine receptor ligands with many other targets, including G protein-coupled receptors, transporters, enzymes, and ion channels, complicates the search for new targets for the treatment of the mentioned diseases [103].

A typical example of such a non-neurological function is, for example, the fact that the dopaminergic system can adapt to various physiological or pathological conditions that the kidneys are exposed to throughout life, maintaining normal blood pressure, insulin resistance, and redox balance. Different dopamine receptors (D1–D5) exhibit a protective effect against hypertension and kidney disease. It is necessary to consider the various interactions of the dopaminergic system with other neurotransmitter systems [104]. In addition, the mesolimbic dopamine system, which originates in the ventral tegmental area and projects to the striatum, is involved in the expression of sex-specific behavior and is considered an important mediator of many mental illnesses. While substantial work has focused on sex differences in dopamine neuron anatomy and the relative dopamine levels in males and females, an important characteristic of dopamine release from axon terminals in the striatum is that it is rapidly modulated by local regulatory sex-independent mechanisms. These processes may occur through homosynaptic mechanisms such as presynaptic dopamine autoreceptors and dopamine transporters, as well as heterosynaptic mechanisms such as retrograde signaling from postsynaptic cholinergic and GABAergic systems, among others. These regulators serve as potential targets for the sex-different expression in dopamine regulation in both ovarian hormone-dependent and -independent manners. The identified sex differences in dopamine function will play an important role in the development of drugs for the treatment of the mentioned disorders in both sexes [105].

## 4. Cortisol-TAT-Mediated Dopamine Regulation

The activity of the enzyme tyrosine aminotransferase (TAT) in humans and animals is determined by various regulatory factors. These include primarily hormonal levels, diet, and age [106,107].

Brook trout fed a high-protein/low-carbohydrate diet had higher TAT activity than fish fed a low-protein/high-carbohydrate diet. Importantly, high cortisol levels increased TAT activity in the liver [108,109]. The authors of the article conclude that TAT activity in the liver of brook trout correlates with protein catabolism, given the greater amount of incoming tyrosine, omitting an important carbohydrate component of the experimental diets that activates dopamine systems, and, as a result, changing the ratio of catabolic and anabolic processes.

It is known that the TAT enzyme has several isoforms, some of which have a high affinity for α-ketoglutarate. In addition, some TAT isoenzymes can use oxaloacetate as an amino group acceptor which, in the case of a high level of activity of this enzyme, can affect the rate of catabolic reactions, for example, through the malate–oxaloacetate shuttle, which carries out a transport function followed by the oxidation reaction of malate to oxaloacetate in the mitochondria under the action of malate dehydrogenase. In this case, oxaloacetate is transferred back to the cytoplasm from the mitochondria after transamination into aspartate using aspartate aminotransferase [110].

Thus, tyrosine aminotransferase, using various amino group acceptors, can regulate the direction of transamination reactions, which, in turn, leads to a shift in metabolic reactions in one direction or another. Cortisol is a glucocorticoid hormone secreted by the adrenal cortex and controlled by the somatotropin-releasing hormone of the hypothalamus by the synthesis of ACTH influencing the pituitary gland. Cortisol is one of the important regulators of metabolism (primarily protein and carbohydrate metabolism). At the same time, this hormone is secreted during the day in different ways—its minimum concentration is detected in the evening.

Obesity is a characteristic sign of Cushing’s syndrome (hypercorticism). In this regard, a lot of studies have been conducted on feedback, namely, that hypercorticism is a common sign of obesity. Hypercorticism in the body occurs in two forms: systemic hypercorticism, in which there is a general excess of cortisol in the body, and tissue or intracellular hypercorticism, in which there is an elevated intracellular concentration of cortisol without a general excess in the body. Correct assessment of the first parameter requires correction for active metabolic mass. The second parameter can be confused with marked fluctuations in plasma cortisol concentration from moment to moment due to episodic cortisol secretion. In this case, correct assessment requires measurement of the average concentration over 24 h. Of the two parameters of systemic cortisol, the plasma concentration is the most important and diagnostically accurate [111].

In addition, cortisol affects cognitive functions in both healthy individuals and patients with neuropsychiatric disorders, in particular with disorders of dopamine systems. At the same time, a correlation was observed between the functional activity of the dopamine transporter (DAT) in the striatum and cognitive functions. In clinical studies, patients with carbon monoxide poisoning were predictably found to have lower cognitive abilities compared to healthy individuals. In all participants, plasma cortisol levels and DAT expression in the striatum were negatively and positively associated with cognitive functions, respectively, including memory and executive function [112]. Another study showed that high stress levels, and, as a result, higher concentrations of glucocorticoids in the blood, increase the level of mesolimbic dopamine (DA) [113]. To test the hypothesis that glucocorticoid responses to psychological stress correlate with DA and subjective responses to psychostimulants in humans, half of the volunteers were given intravenous saline and the other half were given an amphetamine-containing drug before the study. Results showed that stress-induced cortisol levels were positively associated with amphetamine-induced dopamine release in the ventral and other striatal regions. Volunteers with higher cortisol responses to stress also reported more positive subjective effects of amphetamine-containing drugs than volunteers with lower responses. These results indicate a link between glucocorticoids and activation of the mesolimbic dopamine system under conditions of high-stress load [113]. Thus, an increase in the concentration of cortisol activates tyrosine aminotransferase, which metabolizes tyrosine via the non-dopamine pathway, reducing the total amount of tyrosine in the body and thereby affecting the synthesis of dopamine in the brain. A decrease in the concentration of dopamine (including through the projection of dopaminergic neurons to the hypothalamus) leads to a decrease in catabolic and the activation of anabolic processes. At the same time, the amount of aspartate, the activity of AST, and, as a consequence, the flow of oxaloacetate into the tricarboxylic acid cycle decreases. This activates the glucose-alanine cycle (Cahill cycle) in the liver, an increase in the concentration of alanine and pyruvate occurs, and ALT activity increases, which, in turn, leads to a metabolic shift towards gluconeogenesis. It is important to note that the physiological effect of glucocorticoids on dopamine systems is multidirectional and dose-dependent—a moderate increase in the level of these hormones activates tyrosine aminotransferase, reducing the level of tyrosine (a precursor of dopamine), and, as a result, inhibiting dopamine systems, and high levels observed in serious stressful situations, on the contrary, can lead to activation of the mesolimbic dopamine system. These biological effects occur through various hormone-dependent regulatory mechanisms and depend on the initial concentration of glucocorticoids in the blood, and the type and duration of stress exposure.

## 5. The Role of Aminotransferases in Metabolic Regulation and the Diagnostic Role of the De Ritis Ratio

### 5.1. Aspartate Aminotransferase (AST)

Aspartate aminotransferase (2.6.1.1) is an enzyme of the transferase group that catalyzes the reaction of aspartic acid (aspartate) conversion to oxaloacetic acid (oxaloacetate). It is important to note that this reaction can proceed in both directions. In humans, the *GOT1* and *GOT2* genes are isolated, encoding two AST isoenzymes that exhibit activity in the cytoplasm and mitochondria, respectively [114]. The use of AST as a diagnostic marker of blood biochemistry consists of assessing the activity of this enzyme in blood plasma which shows serious pathological disorders in the functioning of the heart and liver, such as myocardial infarction and liver cirrhosis (in fact, the degree of cytolysis of the cells of these organs), but does not reflect the main neurometabolic biological function of this enzyme.

For a better understanding of the mechanisms of action of AST and its two substrates/products, aspartate and glutamate, in the dopamine/cAMP/CREB-induced transcription of genes of the neurometabolic regulation of metabolic processes, we will consider several important results of studies of the brain and liver. cAMP can stimulate the transcription of many GPCR-dependent genes by activating the transcription factor CREB—cAMP response element binding protein [115]. However, in cultured mouse cortical neurons before synaptogenesis, neither cAMP nor dopamine, which acts via cAMP, stimulated CREB-dependent gene transcription when NR2B-containing NMDA receptors (NMDARs) were blocked.

Stimulation of transcription by cAMP was enhanced by excitatory amino acid uptake inhibitors, suggesting a role for extracellular glutamate or aspartate in cAMP-induced transcription. Aspartate was identified as an extracellular messenger; enzymatic removal of L-aspartate, but not glutamate, blocked stimulation of CREB-dependent transcription by cAMP.

Moreover, cAMP induced the release of aspartate but not glutamate. Taken together, these results suggest that cAMP acts through an autocrine or paracrine aspartate release pathway that activates NR2B-containing NMDARs, resulting in Ca^2+^ influx and transcriptional activation. This cAMP/aspartate/NMDAR signaling pathway may mediate the effects of neurotransmitters such as dopamine on axon growth and synaptogenesis in developing neurons or on synaptic plasticity in mature neuronal networks [115]. Glutamate and GABA are the major excitatory and inhibitory neurotransmitters in the CNS, respectively. In chick retinae, GABA is localized to horizontal and amacrine cells, as well as to some ganglion cell layers. Glutamate and its agonists, NMDA, kainate, and aspartate, have been shown to promote GABA release from isolated retina and cultured retinal cells. Dopamine, the major catecholamine in the retina, inhibits the induction of GABA release by NMDA. Thus, NMDA and aspartate induce GABA release exclusively from retinal amacrine cells, and this release is modulated by dopamine. On the other hand, kainate stimulates GABA release from both amacrine and horizontal cells without the intervention of dopamine [116].

Since D-aspartate stimulates the release of prolactin and LH, another group of scientists aimed to determine whether D-aspartate alters the release of hypothalamic and posterior pituitary factors involved in the control of their secretion and whether its effects on these tissues are mediated by NMDA receptors and nitric oxide. In the hypothalamus, D-aspartate stimulated the release of luteinizing hormone (LHRH), α-melanocyte-stimulating hormone (α-MSH), and GABA and inhibited dopamine release through interaction with NMDA receptors. It increased nitric oxide synthase (NOS) activity and its effects on LHRH and hypothalamic GABA release were blunted by NOS inhibition. In the posterior pituitary gland, D-aspartate inhibited the release of GABA but did not affect the release of dopamine or α-MSH. Thus, D-aspartate differentially affects the release of hypothalamic and posterior pituitary factors involved in the pituitary hormone secretion regulation [117].

Dopamine neurons in the substantia nigra pars compacta regulate not only motor but also cognitive functions. NMDA receptors play a crucial role in modulating the activity of these cells. Given that the amino acid D-aspartate has recently been shown to be an endogenous NMDA receptor agonist, the present study aimed to investigate the effects of D-aspartate on the functional properties of dopamine neurons. A comparison of the electrophysiological effects of D-aspartate in control mice and D-aspartate oxidase gene knockout (Ddo(−/−)) mice, which showed a concomitant increase in brain D-aspartate levels, revealed improvement in synaptic plasticity and cognitive function. The effects of L-aspartate, a known endogenous agonist of dopamine neurons, were analyzed in control and Ddo(−/−) mice. D- and L-aspartate excite dopamine neurons by activating NMDA, AMPA, and metabotropic glutamate receptors. D-aspartate was shown to act on midbrain dopamine neurons, activating not only NMDA but also non-NMDA receptors. Dopamine neurons under conditions of high D-aspartate levels create a protective mechanism by compensating for the increase in NMDA receptors and cellular hyperexcitability, which may prevent subsequent hyperdopaminergia in target areas that may lead to neuronal impairment, degeneration, motor, and cognitive impairment [118]. Cocaine exposure increases extracellular D-aspartate, L-glutamate, and D-serine in the prefrontal cortex. This may indicate that cocaine activates dopamine D1 receptor signaling and the PKA pathway in the prefrontal cortex [119].

Another group of researchers studied the release of endogenous aspartic acid from the rat striatum using synaptosomes [120]. The depolarization-induced release of endogenous aspartate was calcium-dependent and mediated via D2 receptors, which is consistent with the hypothesis that aspartate is released as a messenger from striatal axon terminals. The possibility of a co-release of aspartate and glutamate from these terminals is currently under discussion.

The mitochondrial aspartate–glutamate transporter Aralar/AGC1 is a regulatory component of the malate–aspartate shuttle. Aralar/AGC1 deficiency in mice and humans causes a shutdown of brain shuttle activity and global cerebral hypomyelination. An absence of neurofilament-labeled processes was detected in the cerebral cortex, but it is still unknown whether different neuronal types are differentially affected by Aralar/AGC1 deficiency. Postnatal Aralar knockout (Aralar-KO) mice exhibited hyperactivity, anxiety-like behavior, and hyperreactivity with decreased dopamine (DA) in terminally rich areas. Decreased vesicular monoamine transporter-2 (VMAT2) levels were observed, and this was associated with increased DA metabolism via MAO activity (DOPAC/DA ratio) in the striatum of Aralar-KO. However, no decrease in DA or nigral-tyrosine hydroxylase-positive cells was detected in the brainstem of Aralar-KO. Adult Aralar-hemizygous mice also exhibited an increased DOPAC/DA ratio in the striatum and increased sensitivity to amphetamine. These results show that Aralar deficiency causes a drop in the GSH/GSSG and VMAT2 ratios in the striatum, which may be associated with a failure to produce mitochondrial NADH and an increase in cytosolic reactive oxygen species (ROS). The results indicate that the nigrostriatal dopaminergic system is a target of Aralar deficiency [121].

Sodium-dependent ^[3H]^D-aspartate uptake was measured in rat striatal homogenates. The uptake was inhibited by both L- and D-glutamate, with IC50 values of 5.6 and 224 μM, respectively. Dopamine (10^−7^–10^−4^ M), apomorphine (10^−7^ M), sulpiride 10^−6^ M) or a combination of dopamine and sulpiride were found to not affect the observed ^[3H]^D-aspartate uptake. Thus, in vitro dopaminergic modulation of high-affinity glutamate uptake described in the literature was not detected when using ^[3H]^D-aspartate instead of ^[3H]^L-glutamate [122].

### 5.2. Alanine Aminotransferase (ALT)

Like aspartate aminotransferase, alanine aminotransferase (2.6.1.2) is an enzyme of the transferase group. ALT catalyzes the reaction of converting alanine into pyruvic acid (pyruvate). This reaction, as in the case of AST, can proceed in both directions and in humans, the GPT1 and GPT2 genes are isolated, encoding two ALT isoenzymes that exhibit activity in the cytoplasm and mitochondria, respectively [123]. ALT, like AST, is used as a diagnostic marker of biochemical blood analysis, showing pathological disorders in the functioning of the heart and liver. However, this enzyme has its own important neurometabolic biological function. To better understand the role of ALT and its two substrates/reaction products, alanine and pyruvate, in the dopamine/cAMP/CREB-induced transcription of genes involved in the neurometabolic regulation of metabolic processes, we will review several important research findings. Dopamine D2 receptor agonists, bromocriptine and cabergoline, are important drugs for the treatment of Parkinson’s disease and other functional disorders of the monoamine systems. A link has been found between the development of myocardial infarction and long-term treatment with dopamine D2 agonist drugs identified by partial activation of the 5-hydroxytryptamine 5-HT2A receptor. The study was aimed at investigating the activity of the 5-HT2A receptor blocker sarpogrelate in reducing myocardial injury induced by long-term use of D2 receptor agonists in rats with alloxan diabetes [124]. In another study, inhibition of the D2 receptor by haloperidol reduced the indices of oxidative and fibrotic liver injury via the TGF-β1/Smads pathway and NFκB [125]. Thus, dopamine may be involved in the regulation of diabetic liver injury, but the exact role of DRD2 in the pathogenesis of this process is currently unknown.

Apolipoprotein A-1 (APO A1) levels were increased by haloperidol after 7 days but not after 14-day treatments in rats receiving ethanol orally at a dose of 4 g/b.w. for a week, compared with the control group. High-density lipoprotein levels tended to increase only with ethanol treatment for 14 days. ALT (80%) and AST (43%) levels were increased in the group of rats receiving haloperidol after a week of ethanol compared with the control group [126].

A decrease in the serum branched-chain amino acids (BCAA) to aromatic amino acids ratio (Fisher’s coefficient) reflects the severity of hepatic encephalopathy, and clinical improvement has been demonstrated by dietary BCAA supplementation. Since behavioral changes result from changes in central neurotransmission, we investigated the role of BCAA supplementation in altering central neurotransmitter levels in acute liver injury. Male Wistar rats were subjected to liver ischemia by occlusion of the left portal vein and hepatic artery for 90 min. A 4% BCAA solution containing valine, leucine, and isoleucine was injected intraperitoneally three times (8 mL/kg) at 1 h, 6 h, and 24 h after vascular reperfusion, and changes in extracellular concentrations of neurotransmitter amino acids, monoamines, and their metabolites were assessed in the striatum by microdialysis. Although extracellular dopamine concentrations were not affected by either liver ischemia or BCAA injections, the level of 3,4-dihydroxyphenylacetic acid, a metabolite of dopamine, was reduced to 34% in the ischemic group 24 h after reperfusion. The level of 3,4-dihydroxyphenylacetic acid was normalized by BCAA treatment. Improvement of impaired cerebral dopaminergic activity may be a contributing factor to the amelioration of hepatic encephalopathy by BCAA [127].

Another group of scientists conducted a time- and dose-response study to examine the effects of the substituted hydrazine monoamine oxidase (MAO) inhibitors iproniazid and nialamide on the following parameters: MAO-A and -B activity; levels of gamma-aminobutyric acid (GABA), alanine, and the neurotransmitters dopamine, norepinephrine, serotonin and their acid metabolites; and GABA transaminase and ALT activity. The results showed that these drugs are relatively potent MAO inhibitors; but, unlike the unsubstituted hydrazine, the MAO inhibitor phenelzine, they do not cause an increase in brain GABA and alanine levels. These experiments suggest that the free hydrazine group is necessary for the MAO inhibitors to also have a significant effect on GABA and alanine [128]. It has also been found that excessive peripheral sympathetic activity may play an important role in the immersion stress-induced increase in plasma enzyme activity, primarily through β-adrenergic receptors, while α-adrenergic receptors and cholinergic nerves may be involved in the stress-induced increase in enzyme activities, urea, and glucose levels in the blood [129].

### 5.3. Tyrosine Aminotransferase (TAT)

Tyrosine aminotransferase (2.6.1.5) is the third important enzyme of the transferase group, encoded by the *Tat* gene, and converts tyrosine into p-hydroxyphenylpyruvate by metabolizing tyrosine in the liver via the non-dopamine pathway, which certainly affects the dopamine systems of the brain [130].

**Figure 2:**
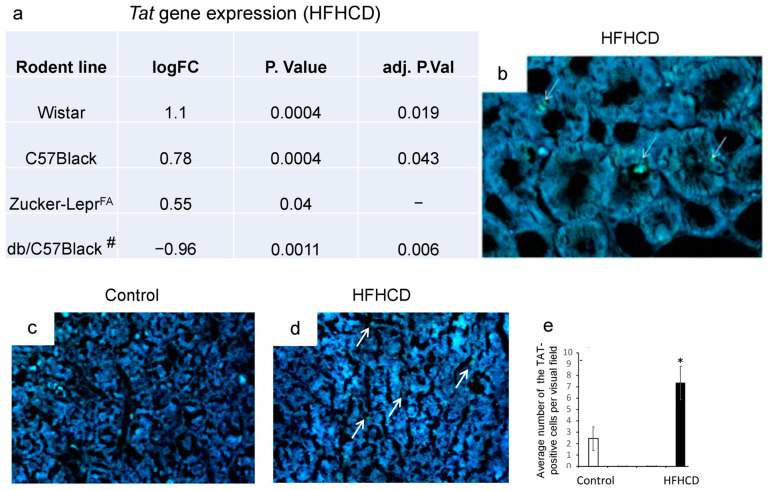
Differential expression of the *Tat* gene (**a**) and immunohistochemical analysis (**a**–**d**) of kidney (**b**) and liver (**c**–**e**) tissue samples from mice and rats of different lines on the background of consumption of a diet with excess fats and carbohydrates (HFHCD) [131,132,133]. Arrows indicate areas of the TAT protein expression. *—*p* < 0.05 compared to the control group; ^#^—control diet.

The differential expression of the enzyme tyrosine aminotransferase, which metabolizes tyrosine via the non-dopamine pathway, found in mice and rats from the groups fed a high-fat, high-carbohydrate diet (HFHCD), may be a consequence of regulatory changes in phenylalanine metabolism in the liver of more metabolically active mice (shown using whole-transcriptome analysis of the liver) in contrast to rats, which showed changes in the differential expression of genes involved in the metabolic pathways of tyrosine but not phenylalanine metabolism [131,132,133]. It is important to note that db/db mice with genetically mediated leptin receptor deficiency showed negative differential expression of the *Tat* gene, and differential expression was also reduced in Zucker rats with the same mutation. Differential expression of the *Tat* gene in all studied lines was observed on the most high-calorie HFHCD, but not on other diets. Thus, whole-transcriptome analysis of liver tissue from Zucker rats and db/db mice (obesity models) revealed the opposite result of differential expression of the Tat gene compared to other rodent strains and hybrids, including inbred C57Black/6J mice, DBCB mice tetrahybrids, and outbred Wistar rats [131,132,133]. The obtained result was confirmed by immunohistochemistry for Wistar rats and revealed a high level of this enzyme in the liver and kidneys of rodents fed a high-calorie diet also (Figure 2) [131].

Thus, tyrosine aminotransferase in the liver plays an important role in tyrosine metabolism via the non-dopamine pathway, which is a new neurometabolic function of the liver aimed at dopamine-mediated regulation of metabolic processes associated with the consumption of high-calorie diets.

This hypothesis is supported by the results of numerous studies indicating that high cortisol levels increase tyrosine aminotransferase activity in the brain and liver. For example, the administration of cortisol to rats increased the activity of tyrosine-2-oxoglutarate aminotransferase (TAT synonym) in the brain [134].

Studies have been conducted on changes in the isoenzyme spectrum of tyrosine aminotransferase in rat liver cortisol induction and during liver regeneration after partial hepatectomy. Electrophoresis in agar gels revealed two groups of tyrosine aminotransferase isoenzymes in the liver. One of them showed a sharp increase in activity after the introduction of cortisol. For the second group, no change in TAT activity was found. When the action of the inducing hormone stops, the activity of the induced isoenzymes drops to the initial level, while the activity of the other group of isoenzymes remains unchanged. An increase in the total level of tyrosine aminotransferase activity after induction with cortisol and partial hepatectomy is the result of an increase in the activity of only some isoenzymes [135].

The concentration of p-tyramine (a metabolite of tyrosine and dopamine) in the striatum of rats increased significantly with intraperitoneal administration of phenelzine [136]. Unlike other monoamine oxidase inhibitors (MAO), phenelzine did not affect the level of p-tyramine in the first 1–2 h after injection and this effect was dose-dependent. In addition, a high dose of phenelzine significantly increased the level of p-tyrosine in the striatum 12 h after injection. Further studies have shown that phenelzine inhibits tyrosine aminotransferase and aromatic L-amino acid decarboxylase activity in rat liver homogenates. Phenelzine also inhibited these enzymes in rat brain homogenates. It is suggested that the multiple enzyme inhibition induced by high doses of phenelzine accounts for its unusual effect on striatal p-tyramine levels compared to other MAO inhibitors. Thus, a decrease in tyrosine aminotransferase activity and an increase in tyrosine concentrations in the striatum of rats were observed under the influence of an MAO inhibitor, which inevitably lead to changes in dopamine levels in this region of the brain.

More than 40 years ago, work was published determining the activity of tyrosine aminotransferase converting factor and further characterizing its kinetic properties. Tyrosine aminotransferase converting factor is a heat-labile substance present in the particulate fraction of rat liver that converts tyrosine aminotransferase form III to form I at 4 °C. Analysis of the distribution of marker enzymes for mitochondria and lysosomes and the converting factor by differential and discontinuous sucrose gradient centrifugation showed that the factor is associated with lysosomes. Converting factor activity was not altered by cortisol administration. Converting factor activity equivalent to that in the liver was also observed in particulate fractions from the kidney and spleen and, to a lesser extent, in the pancreas and salivary gland. No detectable activity was observed in the brain, heart, small intestine, skeletal muscle, red blood cells, serum, or plasma. The presence of converting factor activity in the kidneys and spleen indicates that the substrates for this factor are other proteins, since tyrosine aminotransferase is virtually absent in these tissues [137].

The results of another study showed that when rats were fed a standard semisynthetic diet enriched with fatty acids of varying degrees of saturation, the cortisol-mediated inducibility of the liver cytosolic enzyme tyrosine aminotransferase was altered [138]. Animals fed a diet enriched with saturated fatty acids (in the form of coconut oil) and injected with cortisol intraperitoneally showed a much lower ability to induce tyrosine aminotransferase synthesis than animals receiving a diet enriched with polyunsaturated fatty acids (safflower oil). At the same time, the average values of TAT enzyme activity in response to intraperitoneal injections of cortisol in animals receiving a diet with the addition of corn oil showed an intermediate result.

### 5.4. De Ritis Ratio

#### 5.4.1. AST/ALT-Dependent Regulation of the Energy Metabolism

**Figure 3:**
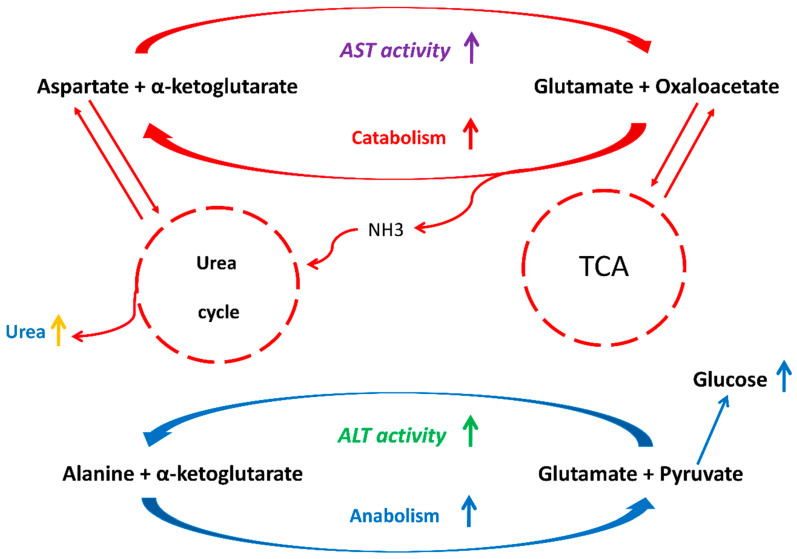
AST/ALT-dependent regulation of the energy metabolism. TCA—trycarboxilic acid cycle.

Several authors note the important role of the De Ritis ratio (the ratio of AST and ALT activities in the blood) in the regulation of metabolic processes in alimentary and other pathological conditions [139,140,141,142,143]. There is very little information on the use of these factors in the functional disorders of monoamine systems diagnosis. The activity of the AST enzyme plays an important role in the regulation of the catabolic and ALT—anabolic reactions. Figure 3 shows the general scheme of AST/ALT-mediated regulation of catabolism and anabolism.

Thus, classical diagnostic indices of various dysfunctions can also be adaptation indices of the normal physiological response of the organism to various environmental influences. On the other hand, the activity of AST and ALT in the blood is often considered an adaptation index of the normal physiological response to any external and internal changes. At the same time, the *Km* of these enzymes in the blood is very low, since the amount of free and available substrates (amino acids) of these enzymes is limited. The change in activity (often within the reference values) of these enzymes is associated with higher metabolic activity of several tissues and organs, including the liver and brain. The same effect demonstrates that the levels of AST and ALT enzyme activity in childhood are higher than in adults, indicating that metabolic changes in childhood occur with greater intensity due to the growth of the organism processes.

#### 5.4.2. Experimental Evidence of the Biological and Diagnostic Role of the De Ritis Ratio in the Pathogenesis of Neurometabolic Diseases

Nevertheless, the important diagnostic role of the De Ritis ratio in the development of metabolic shifts is beyond doubt. Let us examine this using the example of own studies conducted on rats of different lines: the knockout DAT-KO line, with excess extracellular dopamine in the striatum of the brain; Zucker-Lepr^FA^, with a defect in the leptin receptor (obesity model); and the outbred Wistar line, without genetic changes. All rats of the above lines were divided into six groups and received either a control balanced diet or a high fat high carbohydrate diet (HFHCD) [139,144]. The results of the analysis of integral indicators (body weight, relative organ weight, and the main biochemical indicators of blood plasma) revealed differences in rats with the DAT−/− and DAT+/+ (wild type) genotypes for most of the studied indicators. The parameters indicating the high resistance of DAT gene knockout animals to the action of high-calorie diets include the increase in the level of HDL cholesterol in the blood plasma, decrease in body weight, relative weight of the liver, spleen, and white retroperitoneal fat in DAT−/− rats compared to DAT+/+ rats on both experimental and control diets. On the contrary, the level of total protein, albumin, AST, and urea in the blood plasma of DAT−/− was higher than in DAT+/+, regardless of the type of diet, which may indicate a genetically determined effect of excessive (7-fold exceeding the norm) amounts of dopamine in the striatum of the brain of this knockout line of DAT−/− rats on the neuroregulatory function mediating changes in protein and nitrogen metabolism. The latter was manifested in an increased level of AST activity in the blood plasma, which, according to modern data, can be considered not only as an indicator of liver cell damage (accompanied by the release of this enzyme into the systemic circulation) but also as a marker of increased transamination of oxaloacetate with the formation of aspartic acid (aspartate) in the blood plasma, which is a “transport” molecule for the delivery of oxaloacetate to the mitochondria as an activator of catabolic processes in the tricarboxylic acid cycle [139,140,141,142,143,144,145].

**Figure 4:**
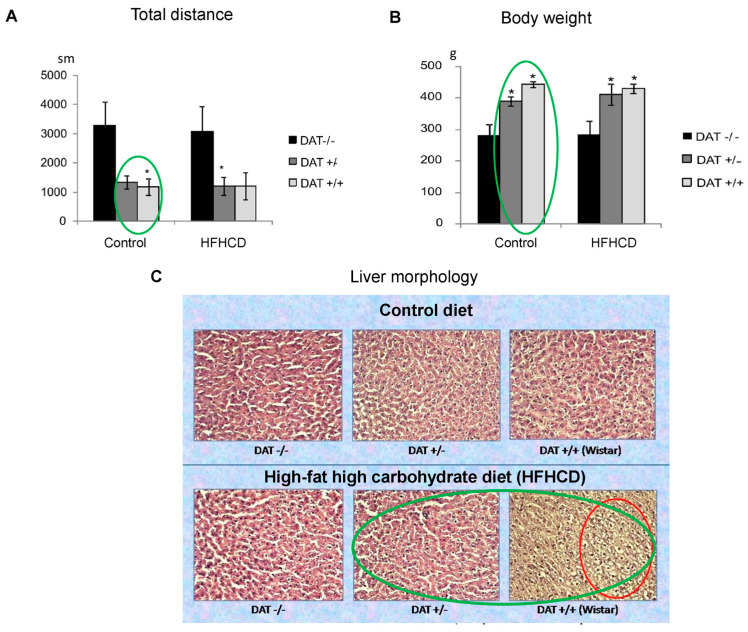
Comparative analysis of locomotor activity. Total distance (**A**), body weight (**B**), and liver morphology (**C**) of the knockout DAT-KO rats’ line, consuming a high-fat, high-carbohydrate diet (HFHCD) [139]. *—*p* < 0.05 in comparison with the control group.

Ammonia, which is a product of amino acid deamination, combines with a carbon dioxide molecule, with the participation of two ATP molecules, turning into carbamoyl phosphate and then into urea in the ornithine cycle. The increased level of total protein, albumin, and urea in the plasma of DAT−/− rats, regardless of the diets used, compared to DAT+/+, confirms this statement. In DAT−/− knockout animals, the increased intensity and lower sensitivity to the action of HFHCD indicate the presence of a central regulation circuit of metabolic processes mediated by dopaminergic neurons of the CNS (Figure 4).

The most characteristic changes and differences in behavioral reactions determined by the knockout of the DAT gene were manifested in rats in the open field test. Impaired dopamine reuptake in DAT−/− homozygotes led to hyperactivity, manifested in the active movement of animals along the perimeter of the arena and higher locomotor activity, as well as an almost complete absence of exits to the central zone of the arena. In contrast, rats with the DAT+/− genotype (heterozygotes) and DAT+/+ (wild type) did not differ in the level of locomotor activity (Figure 4A). At the same time, the body weight of DAT+/− and DAT+/+ rats differed—heterozygotes had a lower body weight compared to the wild type and these differences were statistically significant (Figure 4B). Morphological analysis of the liver parenchyma revealed the absence of fatty liver disease in rats fed a high-fat, high-carbohydrate diet (HFHCD) compared to the control group (Figure 4B) [139].

It is important to note that the extracellular dopamine level in the striatum of heterozygous DAT+/− rats is 1.5–2 times higher than in the control group [146]. At the same time, as stated above, the level of locomotor activity (and therefore the total energy expenditure) of animals in both groups did not differ.

Thus, differences in the level of extracellular dopamine in the brain of rats with the DAT+/- and DAT+/+ genotypes were the key genetic differences leading to a change in the integral and morphological indices of metabolism.

Knockout DAT−/− rats showed a higher level of specific muscle grip strength in comparison with both DAT+/− and DAT+/+ on both types of diets. The obtained result may be associated with the physiological effect of excess dopamine and its metabolites (norepinephrine and adrenaline) in the brain of homozygous rats with the DAT−/− genotype on the activation of fatty acid biosynthesis in adipose tissue, the rate of glucose utilization (catabolism), including muscle and liver tissue (glucose-alanine cycle), and, as a consequence, an increase in the degree of skeletal muscle contraction and an increase in muscle grip strength.

The statistically significant increase in the levels of total protein, albumin, and urea in the blood plasma observed in DAT−/− rats compared to DAT+/+ (wild type), regardless of the diets used, can also be explained by the activation of carbohydrate and alanine catabolism in the glucose–alanine shuttle (Cahill cycle) due to the increased level of total plasma protein (but not ALT activity) and the amino acid pool in the ornithine cycle in the liver under conditions of genetically mediated increased energy expenditure in these animals. At the same time, against the background of HFHCD consumption, the level of urea in the blood decreased in rats of all three genotypes, which can be a manifestation of the suppression of protein catabolism and the urea cycle, due to an increase in the total caloric content of the diet and an increase in the relative proportion of simple carbohydrates (fructose) and fats in the total amount of oxidizable energy substrates. The most noticeable increase in the De Ritis ratio (AST/ALT ratio) in homozygous and heterozygous DAT-KO rats compared to wild-type rats (DAT+/+) against the background of HFHCD consumption indicates an increase in the processes of amino acid catabolism in skeletal muscles, in relation to the liver, under conditions of increased motor activity and associated energy costs caused by the genetic defect in question.

Rats with the DAT−/− genotype initially had increased locomotor activity compared to DAT+/− rats (Elevated Plus Maze test), which was reflected in an increase in the number of visits to open and closed arms of the maze and the total number of transitions between zones. Search activity in DAT+/+ rats was reduced with age, but during the first testing, it was higher than in rats with the DAT+/− genotype and lower than in DAT−/−. At the same time, with age, these differences in DAT+/+ and DAT−/− rats leveled out, which may indicate the influence of dietary fat components on behavioral characteristics and modulation of the transcriptional activity of several genes that determine neuroregulatory processes and feeding behavior. Consumption of HFHCD reduced locomotor activity in DAT−/− and DAT+/+, but not in DAT+/− rats.

Thus, knockout of the dopamine transporter gene (DAT) affects integral indices (increase in the relative mass of the brain, decrease in the relative mass of the liver and spleen, etc.), the state of neuromotor function, the formation of working memory and the level of anxiety, and the consumption of HFHCD, affects the processes of dopamine metabolism in the brain. In addition, in DAT-KO knockout rats, compared to DAT+/+ (wild type), the level of AST, total protein, albumin, and urea in the blood plasma statistically significantly increased regardless of the diets used, which may be due to the activation of carbohydrate and alanine catabolism in the glucose-alanine cycle and the amino acid pool in the ornithine cycle in the liver.

To test this hypothesis and confirm the data obtained, it is necessary to conduct a comparative analysis of the activity of the above aminotransferases and their ratio (De Ritis ratio) in the blood plasma of rats of different lines fed a HFHCD for 2 months.

**Figure 5:**
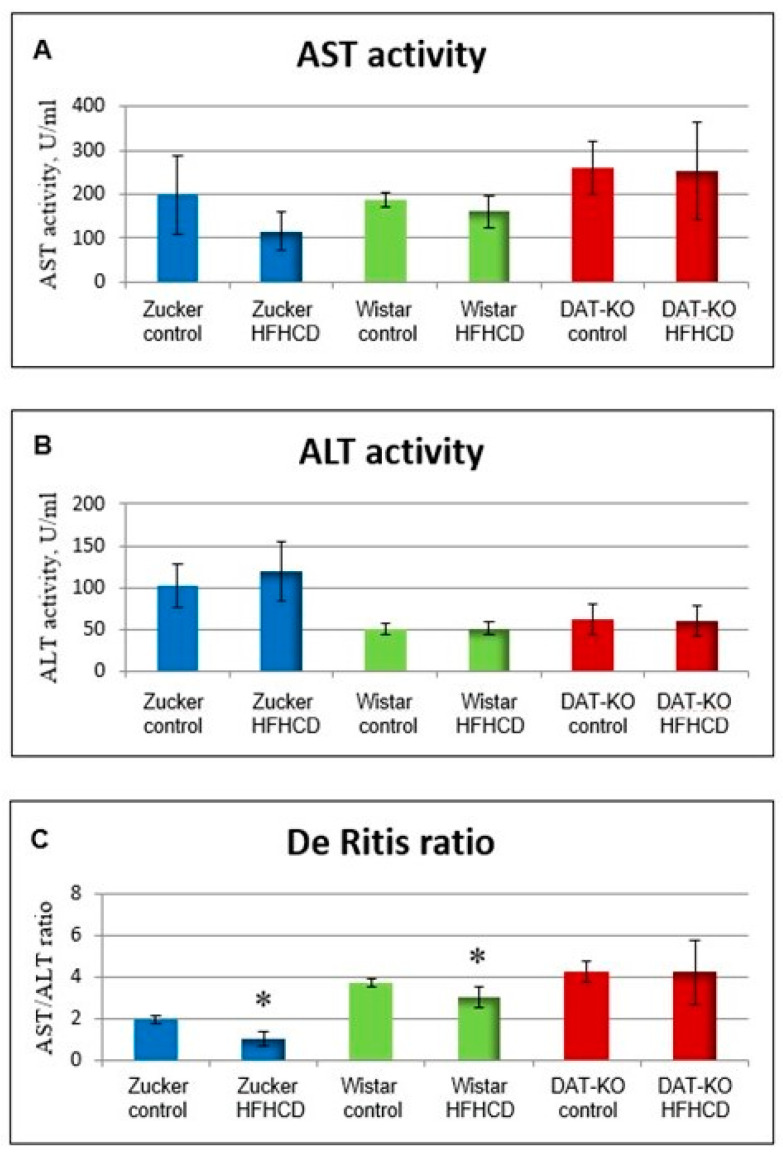
Comparative analysis of the average values of ALT (**A**), AST (**B**) enzyme activity, and De Ritis ratio (**C**) in the blood plasma of Zucker-Lepr^FA^ (obesity model), Wistar, and DAT-KO (hyperactivity model) rats fed a diet with excess fats and carbohydrates (HFHCD) for 2 months. Axis Y (**A**,**B**)—U/mL, axis Y (**C**)—dimensionless value. *—*p* < 0.05 in comparison with the control group.

Figure 5 shows the results of determining the activity of ALT (A), AST (B) enzymes, and the De Ritis ratio (C) in the blood plasma of Zucker-Lepr^FA^ (obesity model), DAT-KO (ADHD model), and Wistar (without genetic changes) rats.

The obtained data show that for all three rat lines, the average values of AST and ALT enzyme activity did not change in the group receiving the HFHCD compared to the control group (within each line). However, the average values of the De Ritis ratio in the blood plasma of Zucker-Lepr^FA^ and Wistar rats receiving the HFHCD decreased (*p* < 0.05), while in DAT-KO rats they did not change, which may be evidence of activation and maintenance of a high level of catabolic reactions under the influence of a high level of extracellular dopamine in the striatum of these animals [139,144]. In addition, homozygous DAT−/− rats were characterized by significantly and repeatedly reduced levels of DA in the striatum in combination with an almost equally increased content of its metabolites—HVA and DOPAC—compared to both heterozygous DAT+/− animals and wild-type rats (DAT+/+, Wistar line) [147]. The effect of the animal line on these parameters was statistically significant according to the two-way ANOVA (*p* < 0.05). Consumption of HFHCD did not significantly affect the levels of DA and its metabolites in rats of all three genotypes. The content of norepinephrine in the striatum of rats of all genotypes receiving the control diet and HFHCD did not differ significantly. Wistar rats are considered the line used in the creation of DAT-KO and are characterized by the greatest similarity to them in the allelic composition of genes, with the exception of DAT. Under these conditions, the only factor influencing the levels of DA and all its metabolites, with the exception of norepinephrine, was the genotype of the animals (allelic type of the DAT gene).

The results obtained are in apparent contradiction with the impairment of DA transport in DAT-KO, leading to the accumulation of this compound in the synaptic cleft. In fact, it should be borne in mind that intracellular and extracellular (in the synaptic cleft) levels of dopamine in the striatum of DAT-KO homozygotes differ.

Dopamine reabsorbed by presynaptic neurons is predominantly accumulated in vesicles for subsequent re-release or is metabolized by monooxygenase with the formation of norepinephrine. In DAT-KO mice, dopamine that accumulates in the synaptic cleft is not reabsorbed but slowly diffuses into the intercellular space, where it can be taken up by astroglial cells expressing biogenic amine transporters that are not affected by DAT knockout. These cells then metabolize DA by monoamine oxidase and aldehyde dehydrogenase to produce increased amounts of DOPAC and HVA.

The consumption of high-fat diets had no significant effect on striatal DA levels and their major metabolites in normal rats, rats with impaired dopamine transport (DAT-KO), and leptin reception (Zucker-Lepr^FA^) [34].

Dopamine deficiency also leads to changes in the hippocampus. The key role of insulin in the hippocampus, where it enhances neuroglial interactions and modulates glucose uptake by astrocytes to meet neuronal energy needs, has been demonstrated [148]. Microdialysis revealed a decrease in DA release into the extracellular space in the rat striatum [149].

**Figure 6:**
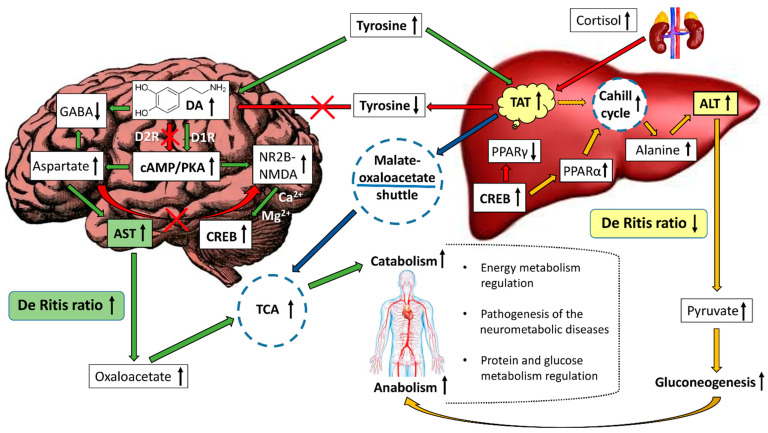
The neurometabolic regulation of energy metabolism by the dopamine–aminotransferase system. Green arrows show biochemical pathways for regulating catabolic reactions, yellow arrows show anabolic reactions, red arrows show the blocking of a biochemical pathway, and blue arrows show multidirectional regulatory function.

Thus, the available data on the effect of an excess caloric diet on DA levels in different parts of the CNS are contradictory, and, in addition, there is no information on the effect of long-term consumption of excess carbohydrates and fat on dopamine metabolite levels in the known studies. In addition, the hypothesis about the association of TAAR1 gene knockout with an increase in the level of catabolic reactions through AST/ALT-dependent dopamine-mediated regulation of protein metabolism was experimentally confirmed [68]. In this study, an increase in the average values of the De Ritis ratio was found in the group of mice with the TAAR1-KO genotype, compared to the control group (wild type) receiving the control diet. At the same time, the average values of the De Ritis ratio in the blood plasma of TAAR1-KO mice did not change depending on the diet, as in the DAT-KO rats from the experiment described above (Figure 6), which indicates the activation of catabolic reactions in both knockout rodent lines. This effect is also evidenced by high relative levels of creatine kinase in the blood plasma of TAAR1-KO mice receiving the control diet and a decrease in body weight in the TAAR1-KO group receiving a high-fructose diet, compared to the wild type [68]. A comparative analysis of the obtained results shows the important role of dopamine and the trace amine system in the regulation of metabolic and behavioral reactions. An important aspect of dopaminergic regulation of the above processes is the effect of DA on the activity of catabolic and anabolic reactions. At the same time, high average values of the De Ritis ratio are observed for the line of homozygous DAT-KO rats, but not Wistar and Zucker-Lepr^FA^, regardless of the diet.

### 5.5. Brain Aminotransferases: Role in Metabolic Regulation

In the CNS, glutamate is an excitatory amino acid. It is stored in presynaptic vesicles and released by calcium-dependent exocytosis. After acting on ionotropic receptors (iGluRs, associated with ion channels) or metabotropic receptors (mGluRs, associated with the metabolic formation of second messengers), glutamate can be removed from the synaptic cleft by two processes: reuptake or diffusion out of the synaptic cleft for uptake by glial cells. This is achieved by glutamate transporters. The level of glutamate available for neurosecretion is regulated by the activity of vesicular transporters. To achieve the correct concentration of neurotransmitters in synaptic vesicles, glutamate must be synthesized. Glutamine is formed in astroglial cells from glutamate reuptake and, since it has no neurotransmitter activity, it is a metabolite that regenerates glutamate in neurons (glutamate-glutamine cycle). In addition, glutamate is also obtained from glucose via an intermediate product of the tricarboxylic acid cycle [150].

Brain glutamate levels change with protein deficiency. These changes are specific to different structures of the brain and depend on protein intake with food or the impact on amino acid metabolism in the central nervous system [151].

Hepatic encephalopathy is a neuropsychiatric disease that develops in patients with severe liver dysfunction and has been known for over a century. However, the pathogenetic mechanisms of cerebral dysfunction associated with liver disease are still poorly understood. The ammonia excess results in hyperactivation of N-methyl-D-aspartate receptors (NMDARs), which in turn affects the aerobic metabolism of the brain, providing it with energy for various biological functions [152].

Further support for the importance of glutamate in brain energy metabolism comes from a study that measured cerebrospinal fluid (CSF) amino acid concentrations and the activity of two transaminases, glutamic oxaloacetate transaminase (AST) and glutamic pyruvate transaminase (ALT), in human Alzheimer’s disease (AD) and a normal brain. It was found that L-glutamic acid, L-glutamine, and L-alanine are the most abundant amino acids in the CSF (50–55% of total amino acids). L-glutamine is found in much higher levels in Alzheimer’s CSF than in normal CSF. In contrast, L-aspartate is found in much lower concentrations in Alzheimer’s CSF than in normal CSF. In the brain with Alzheimer’s disease, AST is present with significantly higher activity than in the normal cerebral cortex (approximately 1.5 times higher). Since the cerebrospinal fluid receives all the necessary amino acids from the brain tissue, and AST catalyzes the conversion of L-aspartate to L-glutamate, higher concentrations of L-glutamine (which is a derivative of L-glutamate) and lower concentrations of L-aspartate are found in the CSF in Alzheimer’s disease, which can be considered a consequence of higher AST activity in the brain [153].

## 6. CREB-Dependent Activation of Neurometabolic Regulation Genes

CREB is a key integrator of various physiological processes in the CNS, including neurotransmission, neurogenesis, neuronal survival, synaptic plasticity, and memory [154]. Dysregulation of CREB signaling is involved in several disorders in the CNS. Dopamine can activate CREB and one of its targets, BDNF, through various biochemical pathways. Deficiency of CREB/BDNF signaling reduces neurogenesis rates and is involved in the development of various brain diseases, including schizophrenia.

Thus, the transcription factor CREB can promote synaptic plasticity and neurogenesis. In addition, there is a relationship between CREB-dependent gene activation, the dopamine system, and neurometabolic regulation of metabolic processes. Let us consider several examples of studies on this topic.

It is known that dopamine can be released by hypothalamic neurons to regulate bone metabolism via the hypothalamic–pituitary–gonadal axis [155]. Neurotransmitter-mediated signals from neurons can act directly on osteoclasts. Dopamine has been shown to inhibit osteoclast differentiation via D2-like receptors (D2R) and CREB during osteoclastogenesis. Dopamine binding to D2R inhibits the cAMP/PKA signaling pathway, which decreases CREB phosphorylation. Pharmacological activation of adenylate cyclase to increase cAMP and PKA production reverses the effects of dopamine. Studies have shown that the D2R/cAMP/PKA/CREB pathway mediates dopamine inhibition of osteoclast differentiation [155]. Another group of scientists showed that dopamine and dopamine D1 receptor (D1R), a member of the dopamine receptor family, play an important role in the progression of hepatocellular carcinoma [156]. The main goal of the study was to investigate the contribution of the dopaminergic system to the pathogenesis of this type of carcinoma and to determine the relationship between D1R and the prognosis of patients with this disease. Dopamine secretion is locally increased due to an imbalance in dopamine metabolism, including activation of dopa decarboxylase and a decrease in monoamine oxidase A. Dopamine promoted proliferation and metastasis against the background of high D1R expression in tumor cells via the cAMP/PI3K/AKT/CREB regulatory pathway, while downregulation of D1R had opposite effects. Moreover, the selective D1R antagonist SCH233900 inhibited in vitro and in vivo tumor cell proliferation and metastasis [156]. Another study demonstrated CREB-mediated tumor growth inhibition and high levels of D3R (D2-like receptor) expression in a hepatocellular carcinoma model [157].

The transcription factor CREB has been shown to play an important regulatory role in normal and pathological liver physiology. Liver fibrosis (LF) is known to be a result of progressive accumulation of extracellular matrix and is a marker of chronic liver diseases. In recent years, numerous studies have contributed to a better understanding of the relationship between CREB and liver fibrosis. Currently, scientists are paying special attention to the study of activation and proliferation of hepatic stellate cells (HSC), cholangiocytes, and the development of antifibrotic therapy [158].

Thus, the results obtained by different groups of scientists indicate an important dopamine-mediated neurometabolic regulatory role of the D1R/D2R/cAMP/CREB biochemical pathway in maintaining normal physiological function of not only the central nervous system but also the liver.

The activation of hepatic stellate cells (HSC) plays an important role in stimulating liver fibrogenesis. Caffeine (the active component of coffee and tea) may reduce the risk of liver disease, including chronic alcohol consumption. One study confirmed the hypothesis that caffeine inhibits the activation of HSCs isolated from a rat model of alcoholic liver fibrosis (AFP) [159]. Rats were gavaged with ethanol to reproduce the AFP model, and then with different concentrations of caffeine or colchicine. Caffeine was found to significantly reduce the levels of ALT and AST in the blood, as well as the overall level of fibrosis in the liver. The results obtained by immunohistochemistry, real-time PCR, and western blotting showed that caffeine has a preventive effect on the development of alcoholic liver fibrosis. The mechanism can generally be interpreted as inhibition of the cAMP/PKA/CREB signaling pathway by caffeine through the A2A adenosine receptor in hepatic stellate cells. Another study in mice examined the effects of caffeine (a non-selective adenosine 2A receptor (A2AR) antagonist) and haloperidol (a selective D2R antagonist) treatment on the fatigue-induced reserpine model of Parkinson’s disease [160]. Reserpinized mice showed impaired motor control in the open field test and fatigue in the grip strength test. L-DOPA and caffeine, together with haloperidol, improved locomotor activity and reduced fatigue. This study showed a positive effect of A2AR antagonism on dopamine signaling through the D2R receptor, which is a consequence of the heterodimerization of both receptor types in the striatum.

An acute dose of 10 mg/kg caffeine causes an increase in the rate of brain glucose metabolism in monoaminergic cell groups such as the substantia nigra and ventral tegmentum, which are rich in dopamine, as well as in medial and dorsal raphe nuclei containing serotonin and the locus coeruleus, rich in norepinephrine. Caffeine increases the metabolic rate in structures of the extrapyramidal motor system and in numerous thalamic nuclei, as well as in the limbic system, such as the hippocampus [161].

It is assumed that the mechanism of this effect is also associated with the cAMP/PKA/CREB signaling pathway not only in the neurons of the striatum, but also in other parts of the brain responsible for neurometabolic regulation of metabolic processes, due to not only the regulation of locomotor activity but also the normalization of the work of AST and ALT aminotransferases in both the liver and the brain. In mice with concanavalin A-induced liver injury, treated with the cannabinoid receptor type II agonist AM1241, a decrease in ALT and AST levels was observed, as well as attenuated liver damage by AM1241, which inhibited the expression levels of *TNF-α*, *IL-6*, and *IFN-γ* genes and proteins in the liver of mice [162]. Phosphorylation levels of p38, JNK, ERK1/2, P65, and CREB proteins were significantly reduced in AM1241-treated mice, while p-JNK levels were increased. The immunohistochemical results were confirmed by Western blot analysis. This study showed that liver injury in mice was protected by the cannabinoid receptor type II agonist AM1241 by modulating immune cells expressing this receptor, such as Kupffer cells. The sympathetic system stimulates while the parasympathetic system suppresses hepatic gluconeogenesis. Insulin stimulates glycolysis and lipogenesis but suppresses gluconeogenesis; glucagon counteracts the action of insulin. Numerous transcription factors and coactivators, including CREB, FOXO1, ChREBP, SREBP, PGC-1α, and CRTC2, control the expression of enzymes that catalyze rate-limiting steps in liver metabolic processes, thereby controlling liver energy metabolism [163].

To study the mechanism of glucagon-mediated regulation of gluconeogenesis, primary hepatocytes from Japanese flounder (Paralichthys olivaceus) were incubated with glucagon [164]. The results showed that glucagon promotes glucose production and increases the mRNA levels of glucagon receptor (Gcgr), guanine nucleotide binding protein α-subunit Gs (Gnas), adenylate cyclase 2 (Adcy2), protein kinase A (PkA), cAMP response element binding protein 1 (Creb1), peroxisome proliferator-activated receptor-γ coactivator 1α (Pgc-1α), phosphoenolpyruvate carboxykinase 1 (Pck1), and glucose-6-phosphatase (G6pc) in hepatocytes. GCGR inhibitor decreased the expression of Gcgr, Gnas, Adcy2, Pka, Creb1, Pgc-1α, Pck1, G6pc mRNA, the expression of phosphorylated CREB and PGC-1α proteins, and glucose production. Overexpression of Gcgr led to the opposite results. PKA inhibitor decreased the expression of Pgc-1α, Pck1, G6pc mRNA, the expression of phosphorylated CREB protein, and glucose production in hepatocytes. CREB inhibitor significantly reduced the stimulation of *Creb1*, *Pgc-1α*, and other gluconeogenesis genes mRNA, and glucose production decreased accordingly. After incubation of hepatocytes with PGC-1α inhibitor, glucagon-activated expression of Pck1 and G6pc mRNA decreased. The obtained results indicate that in Japanese flounder, glucagon promotes gluconeogenesis via the GCGR/PKA/CREB/PGC-1α pathway [164].

Here, a few words should be said about the nuclear transcription factor PPAR, which is a family of receptors (PPARα, PPAR∆ and PPARγ) activated by peroxisome proliferators, present in all organs and tissues of the body and involved in the regulation of metabolic processes and cellular differentiation. PPARα, expressed primarily in the liver, is necessary for metabolic adaptation to starvation, it activates the genes of β-oxidation and ketogenesis. PPAR∆ is actively expressed in skeletal muscles and induces genes responsible for the β-oxidation of fatty acids during starvation and endurance exercise, as well as glucose metabolism and mitochondrial biogenesis. PPARγ is expressed in all tissues, but in adipocytes it is responsible for the activation of genes associated with the synthesis of triglycerides from fatty acids. PPARα and PPARγ play a special regulatory role in carbohydrate and lipid metabolism. For example, the monosaccharide D-arabinose induces expression of the peroxisome proliferator-activated receptor gamma (PPARγ) in the brain, thereby activating CRTC1 transcription via the PPARγ/CRTC1 biochemical pathway. A connection between this process and the pathogenesis of depression has been shown [165].

Glycogen biosynthesis in astrocytes plays an important role in energy metabolism not only in the liver, but also in the brain. Astrocytes, like hepatocytes, sinter glycogen but the precise mechanisms regulating the transcription of this process in astrocytes are still poorly known.

An important regulatory role for PPARγ in regulating metabolic processes has been identified in hepatocytes. Polyethyleneglycol-modified curcumin has been shown to activate CREB, which reduces PPARγ expression in the liver, which may be an effective therapeutic agent to combat steatosis and other diseases induced by high-fat diets [166]. CREB-deficient mice exhibited a fatty liver phenotype and increased PPARγ expression. Moreover, CREB inhibited hepatic PPARγ expression during fasting. Thus, CREB-mediated downregulation of PPARγ expression suggests a potential role for CREB antagonists as therapeutic agents to improve liver insulin sensitivity [167]. 

Vasoactive intestinal peptide (VIP) is known to be involved in the molecular mechanism of initiating glycogen synthesis in astrocytes [168]. HCP-induced glycogen synthesis also requires CREB-mediated transcription, which is dependent on calcium and protein kinase C, but not protein kinase A. In parallel with CREB activation, HCP was shown to also trigger the accumulation of the CREB coactivator CRTC2 in the nuclei of astrocytes. HCP-induced glycogen synthesis has both similar and different molecular mechanisms with glucose-induced glycogen synthesis, including CREB-mediated transcription. Thus, these studies have demonstrated the critical importance of CREB-mediated transcription in astrocytes during glycogenesis [168].

Extracellular magnesium ion (Mg^2+^) is known to be a blocker of NMDA receptors, which play a critical role in the regulation of neuronal plasticity, learning, and memory [169]. Increased extracellular magnesium levels activate CREB via NMDA receptor signaling in cultured rat neurons and brain slices. Extracellular magnesium was also found to activate CREB via NMDA receptors even without extracellular calcium, suggesting a potential independent regulatory role for magnesium in CREB activation. These results indicate that magnesium influx, dependent on NMDA receptor opening, may transduce the signaling pathway for CREB activation in neurons. It is important to note that the relative level of mRNA expression of the transcription factor CREB and the brain-derived neurotrophic factor BDNF in the liver was lower in mice that received regular physical exercise for 6 weeks than in mice in the control group, even though total CREB protein levels were comparable in both study groups [170]. The authors of this study make an important conclusion that increased lactate levels during regular physical exercise enhance its absorption by the liver, changing not only the bioenergetic function of this organ but also limiting the regulation of metabolic processes in the brain. The authors do not specify the mechanisms by which this effect is achieved. At the same time, the relative level of CREB mRNA in the brain did not change.

Thus, dopamine-mediated CREB-dependent activation of a large number of genes leads not only to neurogenesis but also to the neurometabolic regulation of metabolic processes (primarily gluconeogenesis) via NMDA and A2AR receptors and changes in the activities of key transaminases of protein metabolism—AST and ALT.

The general scheme of the regulation of metabolic processes mediated by the dopamine–aminotransferase system is shown in Figure 6.

## 7. Conclusions and Future Directions

In conclusion, we would like to note the complex multi-level physiological neurometabolic system controlled by hormonal and neurochemical signals, as well as enzymatic reactions, the key ones of which in metabolic terms are the transamination of amino (alanine, aspartate, glutamate, tyrosine) and keto acids (oxaloacetate, pyruvate, α-ketoglutarate) using transaminases AST, ALT, and TAT. The key mechanisms of neurometabolic regulation associated with the cortisol-TAT-mediated regulation of dopamine systems, determining the total amount of tyrosine entering the brain, the activation of the transcription factor CREB, and regulating the work of hundreds of genes involved in neurometabolic regulation of metabolic processes in the brain and liver, have been identified. The important role of dopamine in extracellular aspartate level regulation is determined by the stimulation of transcription by cAMP and is enhanced by aspartate and glutamate uptake inhibitors, indicating a role for extracellular glutamate and aspartate in cAMP-induced transcription. Aspartate is an extracellular messenger. However, enzymatic removal of aspartate, but not glutamate, blocks the stimulation of CREB-dependent gene transcription by cAMP. Furthermore, cAMP induces the release of aspartate but not glutamate. Thus, cAMP acts through an autocrine or paracrine pathway of aspartate release, which activates NR2B-containing NMDARs, resulting in intracellular Ca^2+^ entry and CREB-dependent transcriptional activation. Dopamine-mediated aspartate release leads to an increase in AST activity and the activation of catabolic processes.

The increased expression level of the enzyme tyrosine aminotransferase (TAT), which metabolizes tyrosine via the non-dopamine pathway, revealed in mice and rats of various lines fed a high-calorie diet, maybe a consequence of regulatory changes in phenylalanine metabolism (a precursor of tyrosine) in the liver of more metabolically active mice, in contrast to rats which showed differential expression of genes of metabolic pathways of tyrosine metabolism, but not phenylalanine. At the same time, db/db mice with a genetically mediated deficiency of the leptin receptor showed negative differential expression of the *Tat* gene. Thus, tyrosine aminotransferase plays an important role in tyrosine metabolism via the non-dopamine pathway, indicating a new neurometabolic function of the liver aimed at dopamine-mediated regulation of high-calorie diet consumption. It is important to note that tyrosine aminotransferase isoenzymes, using various amino group acceptors, can regulate the directions of transamination reactions, which in turn leads to a shift in metabolic reactions in one direction or another. At the same time, the physiological effect of glucocorticoids on dopamine systems is dose-dependent and reversible—a moderate increase in the level of these hormones activates tyrosine aminotransferase, reducing the level of tyrosine (a precursor of dopamine), and, as a result, reducing the activity of dopamine systems, and the high levels observed in serious stress situations can, on the contrary, lead to activation of the mesolimbic dopamine system. The activity of the AST enzyme plays an important role in regulating catabolic reactions in the body, and ALT plays an important role in regulating anabolic reactions. At the same time, even with reference values of the activities of these enzymes in clinical blood samples, the dynamics of the ratio of ALT and AST activities (De Ritis ratio) in the blood is an important diagnostic indicator that determines the direction of metabolic shifts both in the norm and in the pathogenesis of various infectious, alimentary dependent, and other diseases. The values of the De Ritis ratio in DAT-KO knockout rats (ADHD model) did not change, in comparison with Zucker rats (obesity model) and Wistar, which may be evidence of the activation and maintenance of a high level of catabolic reactions under the influence of a high level of extracellular dopamine in the striatum of these animals.

The next important circuit of metabolic regulation is the D1R/D2R-dependent regulation of metabolic processes in the projection of the brain–liver axis. It is carried out by the innervation of the liver by branches (*rr. hepatici*) of the anterior trunk of the ventral vagus nerve (*nervus vagus*) through the mesolimbic pathway, including D1R expression in the nucleus accumbens (NAc) of the striatum and GABA-ergic regulation of neurons of the lateral hypothalamus, influencing the regulation of carbohydrate metabolism. Treatment with dopamine antagonists is associated with insulin resistance and hyperglycemia, while dopamine agonists are successfully used in the treatment of type 2 diabetes. The number of sympathetic axons is significantly reduced in the liver of mice fed a high-fat diet for a long time, indicating that obesity may induce hepatic sympathetic neuropathy in the long term. Summarizing the experimental results and analysis of the current scientific literature, it can be concluded that an increase in D1R-mediated dopaminergic signaling not only reduces GABA activity in various brain regions, including the hypothalamus, affecting the hepatic innervation of the anterior trunk branches of the ventral vagus nerve, but also increases the level of extracellular aspartate, thereby increasing AST activity and activating catabolic processes. Metabolomic analysis of the liver of iMSN-D2R-KO mice with a deficiency in the expression of the dopamine receptor D2R in striatal neurons, in contrast, revealed an increase in the fat mass ratio compared to animals with a normal genotype.

The results obtained in this review are based primarily on the experimental work of the author and other scientific groups on model animals. At the same time, randomized clinical studies of this physiological mechanism have not been conducted in the world.

For the final confirmation of the hypothesis described by the author in this review, clinical studies are necessary. The author hopes that clinic doctors will read this review and become interested in the diagnostic value of the De Ritis ratio and aminotransferase activity to better understand the pathogenesis of neurometabolic dysfunctions.

Summarizing the results of our research and analysis of scientific literature on the topic of neurometabolic regulation of metabolic processes, it is necessary to single out the dopamine–aminotransferase system of metabolism regulation as a separate, independent, regulatory, and diagnostically significant biochemical pathway controlled by the hormonal system; the key hormone is cortisol, the key neurotransmitter is dopamine, the key transcription factor is CREB, and the key regulatory enzymes are AST, ALT, and TAT.

Thus, the results of the conducted studies indicate a new regulatory mechanism of the neurometabolic physiological function of the human and animal organism, mediated by the dopamine–aminotransferase system.

## Data Availability

Not applicable.
